# Malaria amongst febrile children: call for a pediatric malaria assessment tool

**DOI:** 10.11604/pamj.2021.40.84.21165

**Published:** 2021-10-07

**Authors:** Mumbere Hangi Stan, Megan Andrea Singh, Sejal Paresh Doshi, Susan Andrea Bartels

**Affiliations:** 1 Department of Pediatrics, HEAL Africa Hospital, Goma, Democratic Republic of the Congo,; 2 School of Medicine, Queen´s University, Kingston, Canada,; 3 Departments of Emergency Medicine and Public Health Sciences, Queen´s University, Kingston, Canada

**Keywords:** Children, Democratic Republic of Congo, malaria, pediatric, tool

## Abstract

In 2017, malaria accounted for 435 000 deaths worldwide. Eleven percent (11%) of these deaths occurred in the Democratic Republic of Congo (DRC), where malaria continues to be a leading cause of morbidity and mortality. Children are amongst the most vulnerable to malaria, which causes 40% of childhood deaths in the country. Although many risk factors for developing malaria have been identified, there is a paucity of data available on the sociodemographic risk factors for pediatric malaria. A cross-sectional study including 131 febrile children aged 2 months to 14 years presenting to Heal Africa Hospital due to febrile illness. Guardians of participants answered a questionnaire about household and maternal characteristics, as well as child symptomatology. Malaria status was confirmed via blood smear. Results were analyzed using the chi-square test, likelihood ratios and a logistic regression. The absence of father as head of household (p=0.011) and gestational malaria (p=0.044) were significantly associated with pediatric malaria. This study provides insight into sociodemographic risk factors associated with pediatric malaria in the DRC. While further investigation is required, this study highlights the benefit of considering these factors when approaching the febrile child. A pediatric malaria assessment tool incorporating socio-demographics, symptoms and physical exam findings may guide investigations to reduce unnecessary testing and provide better patient-centred care.

## Introduction

Malaria is one of the most common infectious diseases in the world, accounting for 219 million new cases and 435 000 deaths worldwide in 2017 [1]. While malaria has been confirmed in 87 countries, 90% of cases occur in sub-Saharan Africa [1]. The Democratic Republic of Congo (DRC) accounts for 11% of cases worldwide, second only to Nigeria [2]. Endemic to DRC, *Plasmodium falciparum* malaria is a significant cause of morbidity and mortality. A majority of the population in DRC lives in high transmission zones, with malaria accounting for 40% of childhood outpatient visits and 40% of childhood mortality [2].

Recent changes in funding priorities by the Government of DRC and international investors have led to significant improvements to the Congolese healthcare system [3]. Mortality rates for children under 5 have decreased from 148 to 104 deaths per 1,000 live births and vaccination rates of children ages 12 to 23 months have increased to 45% [3]. However, thirty years of political, security, and economic turmoil have significantly impacted the healthcare system and only 30% of the Congolese population currently have access to health services [3]. Even when access is possible, the healthcare system is often understaffed, under-resourced and, for many, unaffordable [3]. Given these barriers, delivery of care that is efficient, effective and financially sustainable is paramount.

Many risk factors for pediatric malaria have been identified: younger age, presentation to health centres instead of referral hospitals, malnutrition, lack of bed net use, absence of cupboard/radio/bicycle in the home, intake of Artemether-lumefantrine within the preceding two weeks and presence of Chloroquine in plasma [4, 5]. However, there is a paucity of data on the sociodemographic risk factors for pediatric malaria in DRC. Anecdotally, every febrile child who presents to hospitals in DRC is tested with a blood smear to assess for malaria, regardless of risk factors or associated symptoms. Adequate risk factor stratification with clinical consideration can decrease unnecessary testing and empower physicians to select fewer, more high yield investigations. Therefore, the current study aims to elucidate the sociodemographic factors of malaria amongst febrile children at HEAL Africa Hospital in DRC.

## Methods

A cross-sectional case-control study including 131 children aged 2 months to 14 years evaluated in outpatient clinics, the emergency department or admitted to the pediatric ward at HEAL Africa Hospital on weekdays between 8: 00 to 15: 30 from June to November 2018. The sample size was estimated using the Kish formula:


$$N=\frac{Z^{2}P\left(1-P\right)}{D^{2}}$$

Z - standard normal variant corresponding to the 95% confidence interval; P - prevalence of malaria in DRC, 20% [6]; D - the accepted absolute error is 0.05; and N - sample size.


$$\text{Therefore},N=\frac{1.96^{2}0.2\left(1-0.2\right)}{0.05^{2}}=245.7$$

An additional 20% was included in anticipation of possible incomplete data. Therefore, the sample size was calculated to be 295 febrile children. While 300 participants with the chief complaint “fever” were interviewed, 169 were excluded due to absence of a tympanic temperature greater than >38°C at presentation.

**Inclusion criteria:** age <18 years old; febrile (tympanic temperature >38°C [7]) at time of presentation and parental consent obtained

After obtaining informed consent, a structured questionnaire was administered by research personnel, which included two pediatricians, a family medicine resident and a senior medical student at the HEAL Africa Hospital. Four categories of information were included: sociodemographic factors, household factors, child parameters and objective findings. Guided by current literature, these categories were intended to asses socio-economic status, malaria risk, symptoms and signs of malaria as well as medication use [4, 5, 7, 8]. The questionnaire was administered verbally to reduce misinterpretation and mitigate literacy as a barrier. Responses were recorded on paper questionnaires which were entered weekly into Microsoft Excel. Regular discussion between research personnel on survey interpretation allowed for Inter-rater reliability reliability.

Statistical analysis of the data was conducted using Chi-squared analysis, likelihood ratios and logistic regression to determine if there was a statistically significant association between variables and a positive diagnosis of pediatric malaria. As outlined in Table 1, sociodemographic factors, maternal factors, and presenting symptoms and signs associated with pediatric malaria were analyzed. Analysis was conducted in SPSS Version 25.

**Table 1:** baseline characteristics, presenting symptoms and sign of febrile children with or without malaria

	Malaria n (%)	No Malaria n (%)
Number of participants	77 (58.8%)	54 (41.2%)
Mean age (months)	33±32	34±33
**Household factors**		
Antimalarial use for present illness prior to index visit	7 (9.1%)	2 (3.7%)
Bed net use	53 (68.8%)	36 (66.7%)
Insecticide treated bed net	22 (28.6%)	16 (29.6%)
Family size >5 members	62 (80.5%)	45 (83.3%)
Head of household that is not the father	23 (29.9%)	6 (11.1%)
Temporary house	33 (42.9%)	23 (42.6%)
Standing water in neighbourhood	10 (13.0%)	4 (7.4%)
**Maternal factors**		
Mean maternal age	30±5.5	31±5.9
Maternal education > secondary	48 (62.3%)	31 (57.4%)
Unmarried	3 (3.9%)	4 (7.4%)
HIV	2 (2.6%)	1 (1.9%)
Malaria during pregnancy*	30/61 (49.2%)	13/44 (29.5%)
**Child symptoms & physical exam**		
Headache	15 (19.5%)	10 (18.5%)
Chills	30 (39.0%)	8 (14.8%)
Vomiting	37 (48.1%)	11 (20.4%)
Joint Pain	40 (51.9%)	20 (37.0%)
Diarrhea	19 (24.7%)	9 (16.7%)
Pallor	3 (3.9%)	0
Jaundice	0	0
Splenomegaly	0	0
Hepatomegaly	0	0
Temperature at triage	38.7±0.6	38.6±0.5

* Several mothers were unsure about their malaria status during pregnancy, thus they were not included in this analysis

**Primary outcome:** socio-demographic factors that increase pre-test probability of malaria amongst febrile children at HEAL Africa Hospital.

**Ethical considerations:** ethical clearance for this study was obtained from the Institutional Review Board of Heal Africa Hospital. Informed consent was obtained from the guardians of all participants in their preferred language and participation was voluntary without compensation. Study specific serial numbers were employed and surveys were stored in a locked drawer. Data were entered into an Excel spreadsheet on password protected computers to protect confidentiality. All patients who tested positive for malaria were immediately treated with the Congo protocol for malaria treatment [8], in collaboration with the clinical team at Heal Africa Hospital.

## Results

In total, 300 children and their parents were surveyed, of which 131 were included in the present analysis. Table 1 provides a summary of baseline characteristics and presenting symptoms and signs of children with and without malaria. Two historical risk factors were significantly correlated with pediatric malaria: absence of father as head of household (p=0.011) and gestational malaria (p=0.044). As well, two physical exam findings were significantly correlated with pediatric malaria: chills (p=0.003) and vomiting (p=0.001). Please see Table 2 for likelihood ratios of each of these findings. On logistic regression analysis, inclusion of head of household, gestational malaria, chills and vomiting correctly predicted malaria status 70.2% of the time.

**Table 2:** predisposing factors to pediatric malaria

Predisposing Factor	Likelihood-Ratio of Chi-Squared Test
Non-father headed household	6.927
Gestational malaria	4.143
Chills	9.517
Vomiting	10.918

## Discussion

The primary focus of this study was to examine factors that increase the pre-test probability of pediatric malaria amongst febrile children. In total, 131 febrile children who were interviewed and assessed with a blood smear, of which 77 were positive for malaria and 54 were negative, were included. A significant association of pediatric malaria with both chills and vomiting was found, as well as a possible association of pediatric malaria with both head of household as well as gestational malaria.

The present study both supports existing literature and proposes new risk factors for pediatric malaria. The finding of chills and vomiting as signs of pediatric malaria are consistent with current literature, which also includes fever, vomiting and cough [7]. Hepatomegaly, splenomegaly and prostration are considered rare or signs of severe malaria [7]. This study also found a significant correlation between gestational malaria during index pregnancy and pediatric malaria in offspring. To the best of our knowledge, gestational malaria has not been previously described as a risk factor for pediatric malaria, with the exception of congenital malaria [9]. Interestingly, only one of the thirty malaria positive children whose mother had gestational malaria was a neonate. Maternal socioeconomic status and housing characteristics were not collected for the pregnancy period. Perhaps the mother´s environment while pregnant pre-disposed her to malaria, and then the same environment subsequently pre-disposed her child to malaria. The relationship between gestational malaria and pediatric malaria is thus an area that requires further investigation.

This study also revealed a potential sociodemographic risk factor for pediatric malaria: having a head of household that was someone other than the father. Culturally, the father is most often the head of household in DRC. Thus, we must consider its implications on family income (ex. single income families). Interestingly, a similar finding was described in a study conducted in Rwanda, where it was attributed to socioeconomic status [5]. The effect of head of household on the risk of developing pediatric malaria is a significant finding that merits further investigation with careful attention to confounding socioeconomic and demographic factors.

This study is not without limitations. Because blood smears were used without confirmatory polymerase chain reaction (PCR) testing, our outcome measurement relied on the experience of laboratory technicians to read the blood smears, which introduced a potential user dependent error. Thus it is possible that false positives and/or false negatives introduced misclassification bias [10]. Initially, data was collected from 300 participants, however only the 131 patients who were febrile at the time of presentation were included in the analysis, causing a potential selection bias. Anti-pyretic use prior to presentation was not collected, which may further exacerbate the selection bias as patients whose fevers were controlled at triage were not included. Despite a plan to include all children <18 years old, after excluding participants with undocumented fever, only children <15 years old remained. While this may be reflective of the frequency of febrile presentation based on age, it is also possible that the data collection window caused selection bias as it was during school, weekdays and regular work hours. Furthermore, the study was conducted at a single centre in the DRC and is not representative of the DRC pediatric population more broadly.

## Conclusion

This study identified gestational malaria and absence of father as head of household as risk factors for malaria among febrile children. While further investigation is needed, the identification of sociodemographic risk factors should be prioritised in order to better predict malaria risk in febrile children. Furthermore, this study calls for the development of a pediatric malaria assessment tool that considers sociodemographic risk factors, symptoms and physical exam findings to determine the pre-test probability of malaria among febrile children. This tool would guide investigations and improve resource utilization while providing more patient-centred care.

### What is known about this topic

- Known risk factors for pediatric malaria: decreasing age, attendance at health centre instead of referral hospital, low mid upper arm circumference, no bed net use, absence of cupboard, absence of radio, absence of bicycle, intake of Artemether-Lumefantrine within the preceding two weeks and presence of Chloroquine in plasma [4, 5];
- Already known signs of pediatric malaria include fever, vomiting, chills and cough with hepatomegaly, splenomegaly and prostration as more rare/severe signs [7];
- Microscopic examination via blood smear is the gold standard for the detection of malaria. However, this method is user-dependent, relying on the level of expertise of the microscope operator [10].

### What this study adds

- Supports existing research on the signs of pediatric malaria and link between untraditional family structure and pediatric malaria;
- Suggests a correlation between gestational malaria and pediatric malaria;
- Calls for development of a pediatric malaria assessment tool for use in guiding investigation of febrile children in areas with endemic malaria.
